# Comparative Secretome Analysis of *Trichoderma reesei* and *Aspergillus niger* during Growth on Sugarcane Biomass

**DOI:** 10.1371/journal.pone.0129275

**Published:** 2015-06-08

**Authors:** Gustavo Pagotto Borin, Camila Cristina Sanchez, Amanda Pereira de Souza, Eliane Silva de Santana, Aline Tieppo de Souza, Adriana Franco Paes Leme, Fabio Marcio Squina, Marcos Buckeridge, Gustavo Henrique Goldman, Juliana Velasco de Castro Oliveira

**Affiliations:** 1 Laboratório Nacional de Ciência e Tecnologia do Bioetanol (CTBE), Centro Nacional de Pesquisa em Energia e Materiais (CNPEM), Campinas, São Paulo, Brazil; 2 Laboratório de Fisiologia Ecológica de Plantas (LAFIECO), Departamento de Botânica, Instituto de Biociências, Universidade de São Paulo, São Paulo, Brazil; 3 Laboratório Nacional de Biociências (LNBio), Centro Nacional de Pesquisa em Energia e Materiais (CNPEM), Campinas, São Paulo, Brazil; 4 Faculdade de Ciências Farmacêuticas de Ribeirão Preto, Ribeirão Preto, São Paulo, Brazil; Institute for Sustainable Plant Protection, C.N.R., ITALY

## Abstract

**Background:**

Our dependence on fossil fuel sources and concern about the environment has generated a worldwide interest in establishing new sources of fuel and energy. Thus, the use of ethanol as a fuel is advantageous because it is an inexhaustible energy source and has minimal environmental impact. Currently, Brazil is the world's second largest producer of ethanol, which is produced from sugarcane juice fermentation. However, several studies suggest that Brazil could double its production per hectare by using sugarcane bagasse and straw, known as second-generation (2G) bioethanol. Nevertheless, the use of this biomass presents a challenge because the plant cell wall structure, which is composed of complex sugars (cellulose and hemicelluloses), must be broken down into fermentable sugar, such as glucose and xylose. To achieve this goal, several types of hydrolytic enzymes are necessary, and these enzymes represent the majority of the cost associated with 2G bioethanol processing. Reducing the cost of the saccharification process can be achieved via a comprehensive understanding of the hydrolytic mechanisms and enzyme secretion of polysaccharide-hydrolyzing microorganisms. In many natural habitats, several microorganisms degrade lignocellulosic biomass through a set of enzymes that act synergistically. In this study, two fungal species, *Aspergillus niger* and *Trichoderma reesei*, were grown on sugarcane biomass with two levels of cell wall complexity, culm *in natura* and pretreated bagasse. The production of enzymes related to biomass degradation was monitored using secretome analyses after 6, 12 and 24 hours. Concurrently, we analyzed the sugars in the supernatant.

**Results:**

Analyzing the concentration of monosaccharides in the supernatant, we observed that both species are able to disassemble the polysaccharides of sugarcane cell walls since 6 hours post-inoculation. The sugars from the polysaccharides such as arabinoxylan and β-glucan (that compose the most external part of the cell wall in sugarcane) are likely the first to be released and assimilated by both species of fungi. At all time points tested, *A*. *niger* produced more enzymes (quantitatively and qualitatively) than *T*. *reesei*. However, the most important enzymes related to biomass degradation, including cellobiohydrolases, endoglucanases, β-glucosidases, β-xylosidases, endoxylanases, xyloglucanases, and α-arabinofuranosidases, were identified in both secretomes. We also noticed that the both fungi produce more enzymes when grown in culm as a single carbon source.

**Conclusion:**

Our work provides a detailed qualitative and semi-quantitative secretome analysis of *A*. *niger* and *T*. *reesei* grown on sugarcane biomass. Our data indicate that a combination of enzymes from both fungi is an interesting option to increase saccharification efficiency. In other words, these two fungal species might be combined for their usage in industrial processes.

## Introduction

The increasing demand for sustainable energy has promoted considerable efforts to replace fossil fuels with biofuels. As the second world’s largest producer and exporter of ethanol from sugarcane, approximately half of Brazil’s fuel supply is produced from renewable energy sources [1]. Currently, Brazilian production relies on the fermentation of sucrose, known as first-generation (1G) bioethanol. If second—generation (2G) bioethanol was commercialized, Brazil could increase bioethanol production by approximately 40% [2]. To reach this level in industrial processes, the obstacle of cell wall recalcitrance must be overcome. Cell wall recalcitrance is a phenomenon directly related to the enormous complexity of the plant cell wall [3]. In the case of sugarcane, de Souza *et al*. [4] proposed a model for the architecture of polymers within the cell walls of the leaf and culm (the stem of the sugarcane) that included the structural complexity of hemicelluloses, such as arabinoxylan, xyloglucan, and mixed-linkage-β-glucans, as well as pectins, such as homogalacturonnan and arabinogalactans. They found that sugarcane tissues are composed of ca. 30% of cellulose and 60% hemicelluloses, with pectins and lignin accounting for the rest of the biomass [4].

The biomass of sugarcane displaying these features is transformed into bagasse, a major residue from the Brazilian agroindustry (280 kg per 1 ton of sugarcane crushed) [5]. Bagasse is obtained from a process that crushes and washes biomass (to obtain sucrose), changing the composition in relation to culm. Bagasse is composed of cellulose (40–50%), hemicellulose (25–35%), and lignin (15–20%) [6, 7], highlighting the fact that a portion of hemicelluloses and pectins are washed out during sucrose extraction. Thus, the compositions of sugarcane culms *in natura* and bagasse are considerably different, with the former displaying higher complexity and proportionally higher levels of soluble polymers that belong to the classes of hemicelluloses and pectins.

Although considerable progress has been made in the saccharification of recalcitrant plant biomass, the cost of 2G bioethanol will not become economically competitive unless the full conversion of lignocellulose biomass can be reached. Complete hydrolysis of cellulose yields glucose, whereas hemicellulose hydrolysis can produce monomers of xylose, arabinose, mannose, glucose and galactose. In order to break down the cell wall and release these monomers, pre-treatment with enzymatic cocktails are necessary prior to hydrolysis, and this step constitutes the majority of the cost in 2G bioethanol processing [8]. A better understanding of the hydrolytic mechanisms and enzyme secretion of polysaccharide-hydrolyzing microorganisms is needed to overcome the cost associated with enzyme pretreatments. In many natural habitats, plant biomass is degraded by a variety of lignocellulolytic microorganisms that work together to break down the recalcitrant structure of lignocellulosic materials. Although bacteria and yeast (the latter more rarely) produce hydrolytic enzymes [9, 10], most enzymes used in commercial cocktails are derived from fungi, such as *Aspergillus niger* and *Trichoderma reesei*, due to their efficiency in producing and secreting a broad range of cellulases and hemicellulases.

*A*. *niger* is industrially used to produce many pectinases [11, 12] and hemicellulases [13, 14]. A sequencing effort reported that *A*. *niger* contains 14,056 genes [15], and it has one of the most remarkable sets of genes encoding hydrolytic enzymes among sequenced fungal genomes. According to the Carbohydrate-Active Enzymes (CAZY) database (http://www.cazy.org/), *A*. *niger* has more than 250 glycoside hydrolases (GHs). Another hyper producer of cellulolytic enzymes is *T*. *reesei* RUT-C30. This strain was obtained from the wild-type strain, QM6a, after three rounds of random mutagenesis, with the aim of increasing cellulase production.

Due to their great potential for producing hydrolytic enzymes, both of these fungi have been the focus of several studies on GH discovery and there is a marked effort to understand the regulation of the expression of genes that encoding them. To date, only one master carbon repression regulator has been described (CreA/Cre1). The *A*. *niger* transcription factor, XlnR (and the *T*. *reesei* orthologues Xyr1), is a major positive transcriptional regulator of xylanases and cellulases encoding genes for this species. In *A*. *niger*, the expression of most cellulases and hemicellulases is co-regulated by the same inducer (xylose), but for *T*. *reesei*, at least four different inducers have been described (xylose, xylobiose, sophorose and lactose) [16, 17]. Several differences in the regulation of GH production between these two fungi have been already described [16, 18, 19], but comparative studies could provide a more comprehensive overview of how these important industrial species sense and produce hydrolytic enzymes.

Although many secretome studies have been performed using *A*. *niger* and *T*. *reesei* [20–24], very few were based on sugarcane culm and/or bagasse [25, 26]. In the present work, these two fungal species were grown on sugarcane biomass with two levels of cell wall complexity: culm *in natura* and pretreated bagasse. The production of enzymes related to biomass degradation was monitored using secretome analyses after 6, 12 and 24 hours. Concomitantly, we analyzed the sugars released in the supernatant. Our experiments demonstrate that both species degrade biomass after 6 hours post-inoculation, but comparative secretome analysis of *A*. *niger* and *T*. *reesei* revealed that it can occur through different mechanisms. This study provides a better understanding of the saccharification process, and it can be used as a basis for the production of optimized enzymatic cocktails.

## Materials and Methods

### Fungi strains and media

The species used in this work were the filamentous fungi *Trichoderma reesei* RUT-C30 and *Aspergillus niger* N402. Both strains were maintained on potato dextrose agar (PDA) at 29°C and 30°C, respectively. The basic culture medium (BCM) (pH 5.5) was composed of 0.05% yeast extract (w/v), 50 mL/L salt solution (6 g/L NaNO_3_, 1.5 g/L KH_2_PO_4_, 0.5 g/L KCl and 0.5 g/L MgSO_4_), 200 μL/L trace elements (10 g/L ethylenediaminetetraacetic acid, 4.4 g/L ZnSO_4·_7 H_2_O, 1.0 g/L MnCl_2_·4H_2_O, 0.32 g/L CoCl_2_·6H_2_O, 0.315 g/L CuSO_4_·5H_2_O, 0.22 g/L (NH_4_)6Mo7O_24_·4H_2_O), 1.47 g/L CaCl_2_·2H_2_O and 1 g/L FeSO_4_·7H_2_O) and a predetermined concentration of carbon source, according to our experimental conditions (see below).

The exploded bagasse was prepared as described by Souza *et al*. [25]. Briefly, sugarcane bagasse *in natura* was treated with 14 kg/cm^2^ water steam, washed exhaustively with distilled water until reducing sugars were not detected by dinitrosalicylic acid (DNS) [27] and dried at 40°C for several days. The culm was ground into particles with a 2-mm diameter, 3 g of culm particles were washed six times with 25 mL of 80% (v/v) ethanol at 80°C for 20 min, washed with distilled water to remove ethanol, and dried. After drying, both sugarcane exploded bagasse (SEB) and sugarcane culm (SC) were sifted on a 600-μm industrial sieve.

### Substrate-based induction conditions

*T*. *reesei* and *A*. *niger* spores were harvested from fresh potato dextrose agar (PDA) plates by adding 1 mL of sterile distilled water. The spore suspensions were inoculated in triplicate to a final concentration of 1 × 10^6^ spores per 30 mL of BCM (pH5.5) in a 250-mL Erlenmeyer flask containing 1% fructose (w/v) as the sole carbon source. *T*. *reesei* and *A*. *niger* spores were grown at 29°C and 30°C, respectively, for 24 hours (*A*. *niger*) on a rotary shaker with agitation at 200 rpm. *T*. *reesei* was also grown on a rotary shaker with agitation of 200 rpm, but it was grown for 48 hours to achieve an initial mycelial mass similar to that of *A*. *niger*. After, mycelia were removed by filtration through Whatman grade 1 filters (GE Healthcare), and they were washed with sterile water and transferred to 30 mL of fresh BCM media (without yeast extract) containing 0.5% of SEB or SC (w/v) as the sole carbon source for 6, 12 or 24 hours. *T*. *reesei* cultures were grown in a controlled environmental growth chamber under constant illumination with white light.

The mycelia and biomass used as carbon sources were harvested by filtration, washed thoroughly with sterile water and quickly frozen in liquid nitrogen for further cell wall monosaccharide composition analyses. The supernatant was stored at -20°C for enzymatic, soluble supernatant sugar and mass spectrometry analyses.

### Fungal growth

Nitrogen content, an indirect measure of fungal growth, was measured based on the Pregl-Dumas’ classical method [28]. The mycelial mass of *A*. *niger* and *T*. *reesei* grown on BCM with bagasse or culm for 6, 12 or 24 hours was rinsed with distilled water to remove traces of medium, and it was dried at 80°C for 4 h. The sample was macerated, and 2 mg (weighed with a digital electronic balance) was burned at approximately 975°C in the presence of pure oxygen. The process released nitrogen, carbon dioxide and water, which were passed through special columns that absorbed the carbon dioxide and water. A column carrying a thermal conductivity detector separated the nitrogen from any other residue, and the resulting nitrogen content was measured. The instrument (PerkinElmer, model 2400, series II) was previously calibrated by analyzing a pure standard of known nitrogen, and the amount of nitrogen in each sample was given as a percentage in relation to the initial mass.

### Monosaccharide Analyses in Culture Supernatant

Monosaccharide analysis was performed on the supernatant. Each sample (1.8 mL) was completely dried using a Refrigerated CentriVap Concentrator (LABCONCO), resuspended in 500 μL of sterile deionized water and filtered through a 0.45-U pore size, 13-mm diameter (Durapore, Millex). The samples were subsequently analyzed by HPAEC-PAD on a CarboPac PA-1 column (DX-500 system, Dionex). The elution of carbohydrates occurred in a gradient mixture of water and 200 mM sodium hydroxide at a flow rate of 0.8 mL/min for 50 min. Sugars were identified and quantified by comparing the retention times and ratios of sample peak area to internal standard peak area in relation to ratios determined for external standards using a Chromeleon 6.8 Chromatography Data System software.

### Supernatant preparation and SDS-PAGE analysis

To analyze the secretome profiles of *T*. *reesei* and *A*. *niger*, triplicate supernatants (~90 mL) containing enzymes from each time-point were pooled and clarified by filtration through a 0.22-μm filter (Hydrophlic Millex, Millipore). The clarified supernatant was concentrated using a 3-kDa membrane (Vivaspin 20, GE HealthCare) to a final volume of 200 μL, and 20 μL was separated by 10% SDS-PAGE (110 V, 90 min). Three independent biological replicates of pooled supernatants were performed for the secretome experiments. The proteins were visualized by staining with 0.1% Coomassie Brilliant Blue R250 (w/v), followed by destaining with 45% methanol and 10% acetic acid solution (v/v). All bands from triplicate SDS-PAGE gels were manually excised, reduced, alkylated and digested in gel with trypsin-modified sequencing-grade reagents (Promega), according to a previously described method [29]. S1 Fig shows SDS-PAGEs with one replicate of each sample, before the concentration step.

### Mass spectrometry and protein identification

Peptides were resuspended in 0.1% formic acid (v/v), and an aliquot (4.5 μL) was analyzed on an ETD-enabled LTQ Velos Orbitrap Mass Spectrometer (Thermo Fisher Scientific) coupled to a nanoflow liquid chromatography column (LC-MS/MS) via an EASY-nLC System (Proxeon Biosystem) through a Proxeon nanoelectrospray ion source. Peptides were separated by a 2–90% acetonitrile gradient in 0.1% formic acid using an analytical column PicoFrit Column (20 cm x ID75 μm, 5-μm particle size, New Objective), with a flow of 300 nL/min over 27 min. The nanoelectrospray voltage was set to 2.2 kV, and the source temperature was 275°C. All instrument methods for the LTQ Orbitrap Velos were set up in the data-dependent analysis (DDA) mode. Full scan MS spectra (m/z 300–1,600) were acquired in the Orbitrap analyzer after accumulation to a target value of 1e^6^. The resolution in the Orbitrap was set to *r* = 60,000. The 20 most intense peptide ions with charge states ≥ 2 were sequentially isolated to a target value of 5,000 and fragmented in the linear ion trap by low-energy CID (normalized collision energy of 35%). The signal threshold for triggering an MS/MS event was set to 1,000 counts. Dynamic exclusion was enabled with an exclusion size list of 500 and exclusion duration of 60 s. The activation Q-value was 0.25 and the activation time was 10 ms.

Data were acquired using the Xcalibur software package, and the raw data files were converted to a peak list format (mgf), without summing the scans, using Mascot Distiller v.2.3.2.0 (Matrix Science Ltd.). The database search was performed against the *Trichoderma* (13,808 proteins) and *Aspergillus niger* (36,414 proteins) from the NCBI database using the Mascot v2.3.02 engine (Matrix Science Ltd.), with carbamidomethylation as a fixed modification, oxidation of methionine as a variable modification and one trypsin missed cleavage. The precursor mass tolerance was set to 10 ppm, and the fragment mass tolerance was set to 0.1 Da. For protein identification, the resulting search data were analyzed in Scaffold 3.5.1 (Proteome Software). The defined parameters were: minimum protein probability of 80%, minimum peptide probability of 90% and unique different minimum peptide of 1. We accepted proteins with up to 10% FDR protein and 5% FDR peptide. CAZymes with only one unique peptide detected were considered in the present manuscript as less confidently quantified than those proteins with multiple peptides detected. All the single protein matches were further checked if the Mascot MS/MS ion score was greater than 25 (significance threshold *p*< 0.05), giving better confidence to protein identification. To access the signal peptide presence, we used the signal peptide prediction program, SignalP version 4.1 (http://www.cbs.dtu.dk/services/SignalP/). The CAZy database (http://www.cazy.org/) was used to classify the identified proteins according to their families.

### Enzymatic assays

Enzymatic activity was determined from the amount of reducing sugar liberated from different polysaccharide substrates by the 3,5-dinitrosalicylic acid (DNS) method [27] using glucose as standard. First, 30 mL of supernatant from the samples induced with SEB or SC for 24 hours was concentrated using a centrifugal concentrator (Vivaspin 20, 10 kDA, GE HealthCare) to a final volume of 5 mL. The supernatant activity was assayed using xylan from beechwood, β-glucan, debranched arabinan from sugar beet, xyloglucan from tamarind and carboxymethylcellulose (CMC) (purchased from Sigma—Aldrich and Megazyme) as substrates at a 0.5% final concentration. Briefly, activity was measured using 50 μL of the substrate solution, and 20 μL of supernatant was diluted in 100 mM sodium acetate buffer (pH 5.5) to achieve a final volume of 100 μL. The reaction was incubated at 40°C for 5 min for xylan and β-glucan and for 10, 70 and 180 min, for xyloglucan, CMC and debranched arabinan, respectively. The reaction was stopped by adding 100 μL of DNS. One unit (U) of enzymatic activity was defined as the amount of enzyme required to release 1 μmol of reducing sugar per minute.

## Results and Discussion

### Saccharification of sugarcane biomass

**Fig 1:**
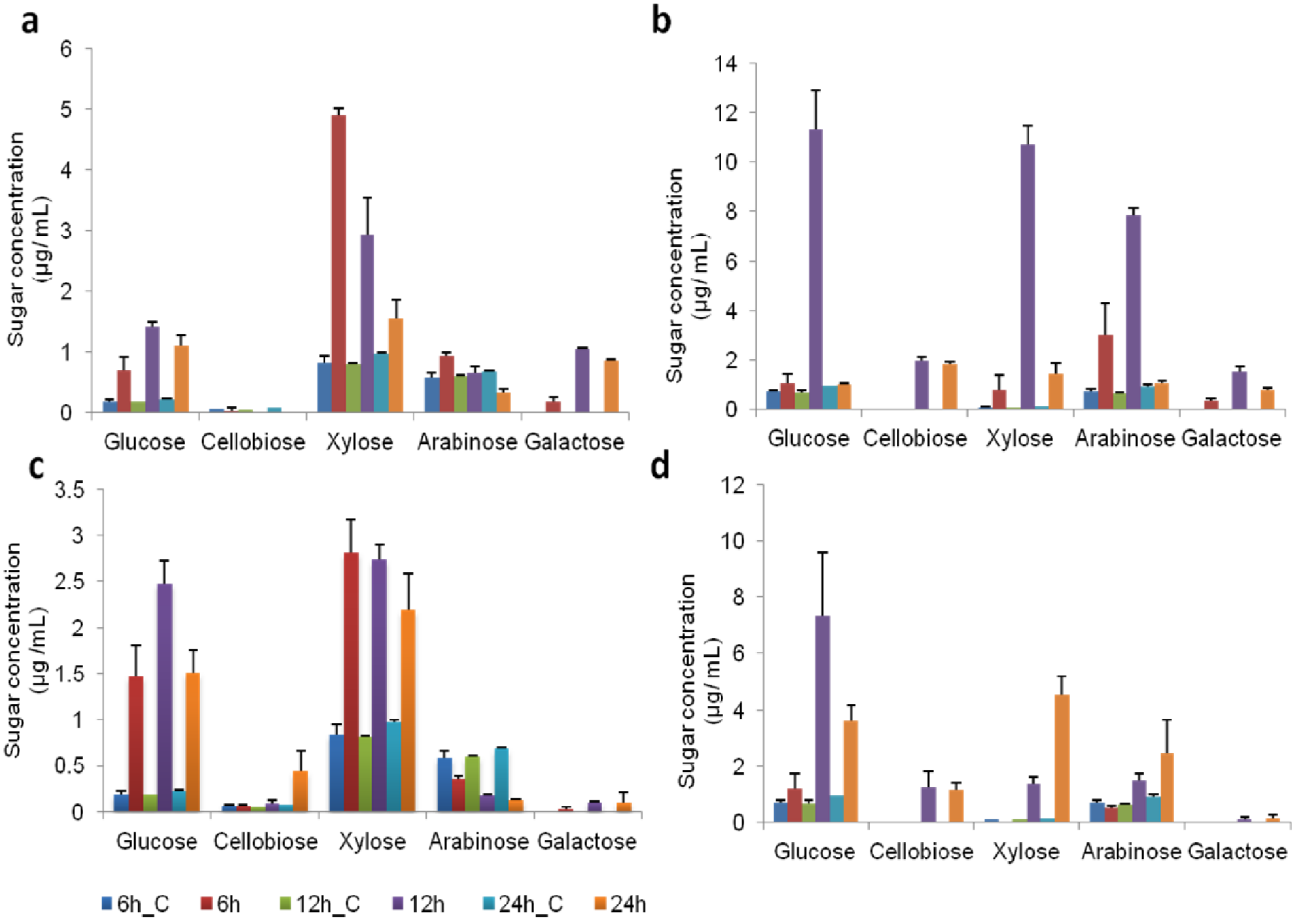
Degradation of sugarcane biomass and the sugars released by *A*. *niger* and *T*. *reesei*. Free sugars in the supernatant after the transfer of mycelia to culm/bagasse media at 6, 12 and 24 hours (h). Each bar represents the mean and the standard deviation of values from three independent experiments. The samples marked C are the control samples that were not inoculated with fungi. a) *A*. *niger* grown on 0.5% culm; b) *A*. *niger* grown on 0.5% bagasse; c) *T*. *reesei* grown on 0.5% culm; and d) *T*. *reesei* grown on 0.5% bagasse.

To identify when the degradation of sugarcane exploded bagasse (SEB) and culm (SC) were initiated, we analyzed the sugars that were released into the *A*. *niger* and *T*. *reesei* culture supernatants using high-performance liquid chromatography (HPAEC-PAD), over a time course of 6, 12 and 24 hours post-inoculation. The supernatant from each time point was also collected from control samples that were not inoculated with fungi. Fig 1 shows the changes in the proportions of glucose, cellobiose, xylose, arabinose and galactose over the course of the experiment. We observed bagasse degradation after 6 hours post-inoculation since the total concentrations of glucose and xylose were higher than in the control samples (Fig 1a and 1c). In culm, this feature was more noticeable after 12 hours (Fig 1b and 1d).

The changes in the proportion of sugars (Fig 1) led us to conclude that both cellulose and hemicellulose were being degraded. We observed only galactose in the samples with fungi that used bagasse and culm as carbon source, suggesting that the degradation of branched hemicelluloses (mainly composed of xylose and galactose) started within 6 hours. It is also likely that the fungi were consuming sugars derived from arabinoxylan, since we detected changes in arabinose concentration. Together with pectin and β-glucan, arabinoxylans form the most water-soluble and accessible part of the cell wall in sugarcane [4]. Therefore, these sugars are readily released by the fungal species as soon as they come into contact with the substrate due the actions of multiple enzymes, such as α-arabinofuranosidases (GH3, GH43, GH51, GH54 and GH62), which remove the residues of arabinose, and β-1,4-endoxylanase (GH10, GH11, GH30) and β-1,4-xylosidase (GH3, GH43), which hydrolyze xylose from the xylan backbone [30].

**Fig 2:**
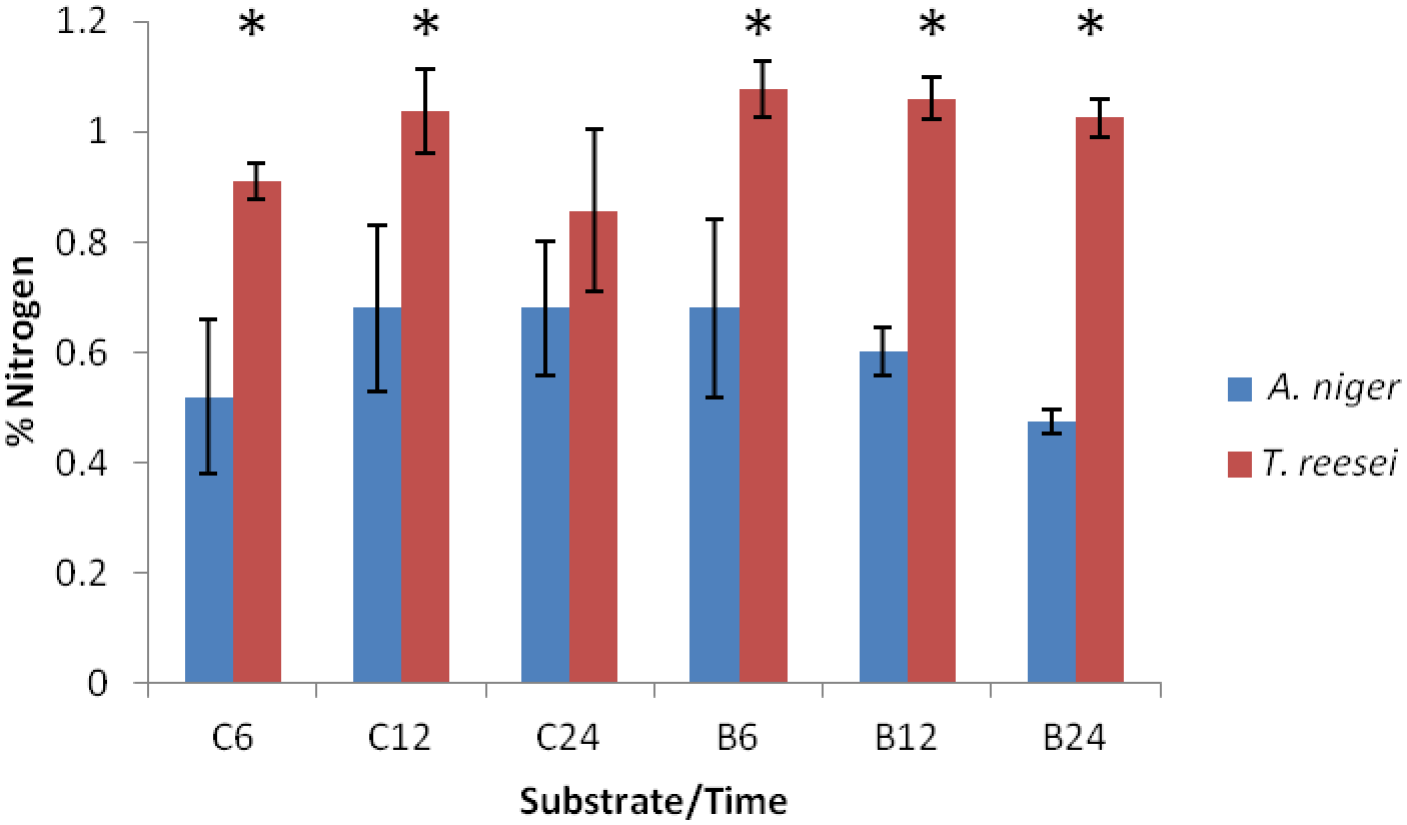
Nitrogen content. Total nitrogen content of fungi growing on sugarcane culm (C) and bagasse (B) for 6, 12 and 24 hours (h). Each bar represents the mean and the standard deviation of values from three independent experiments. The asterisk (*) indicates samples with a significantly different % of nitrogen between fungal species (*p<*0.05) by unpaired t-test, which suggest that *T*. *reesei* has a higher growth at these substrates/time points than *A*. *niger*. There is no difference in nitrogen content among the substrates/time points examined in both species.

Despite the higher recalcitrance of the culm, we also noticed that the samples from fungi grown on this substrate presented the highest amount of sugars in the supernatant (Fig 1b and 1d). These results suggest that both *T*. *reesei* and *A*. *niger* secreted more enzymes using culm as a carbon source compared to bagasse, since there were no differences in growth between the two substrates (indirectly measured by nitrogen content, Fig 2). Because both fungi consume sugars, these values do not represent the real rate of sugar release; however, they suggest that both fungi released different amounts of monosaccharides and/or have different rates of sugar uptake.

### CAZymes profiling of *T*. *reesei* and *A*. *niger* secretomes

Because the sugars released are dependent on secreted hydrolases, we investigated the *T*. *reesei* and *A*. *niger* secretomes. These hydrolysis systems appear depend on the microorganism and carbon source used [23, 31, 32]. To date, few studies have utilized lignocellulosic biomass as a major carbon source, especially with sugarcane biomass [25, 26]. Our data showed that *A*. *niger* secreted a large number of CAZymes at all time points/carbon sources In total, we identified 45 different CAZymes in *T*. *reesei* (Table 1) and 89 in *A*. *niger* (Table 2) (Glycosyl Transferases, GTs, were not included). S1 Table lists all these enzymes and their respective molecular weights, numbers of identified peptides, peptide sequences, mass-to-charge ratios (m/z), numbers of unique peptides, and presence/absence of a signal peptide. S2 Table lists all other secreted proteins (not CAZymes, with signal peptide) found in both secretomes with their respective informations.

**Table 1:** CAZymes detected in the secretome of *Trichoderma reesei*.

CAZy Family	Predicted Protein	JGI Protein ID	Substrate/Time point	Possible Polysaccharide Substrate/Classification
GH17	Candidate glucan 1,3-β-glucosidase	24326	B24h, C6h, C24h	1,3–1,4-β-Glucan
GH17	Candidate glucan endo-1,3-β-glucosidase	110434	C12h, C24h	1,3–1,4-β-Glucan
GH55	Exo-1,3-b-glucanase	25104	B12h, C6h, C12h, C24h	1,3–1,4-β-Glucan
GH55	β-1,3-glucanase	93142	B6h, B12h, B24h, C6h, C12h, C24h	1,3–1,4-β-Glucan
GH1	β-glucosidase CEL1A, bgl2	127115	B12h, B24h	Cellulose
GH3	β-glucosidase, CEL3B	25095	B6h, B12h, B24h, C24h	Cellulose
GH3	β-glucosidase, bgl3i	109567	B24h, C6h, C24h	Cellulose
GH3	β-glucosidase, bgl1, cel3a	136547	B24h, C24h	Cellulose
GH5	Endoglucanase CEL5A	72489	C24h	Cellulose
GH6	Cellobiohydrolase CEL6A, cbh2	122470	B24h, C12h, C24h	Cellulose
GH7	Endoglucanase CEL7B, egl1	5304	C24h	Cellulose
GH7	Cellobiohydrolase CEL7A, cbh1	125125	B6h, B12h, B24h, C6h, C12h, C24h	Cellulose
AA9 (GH61)	Copper-dependent monooxygenase	139633	B24h, C24h	Cellulose
CBM1	Swollenin	104220	B12, B24, C12, C24	Cellulose
GH5	Endo-β-1,4-mannosidase, man5a	122377	C12h, C24h	Mannan
GH79	Candidate β-glucuronidase	69609	C12h	Xylan/Arabinoxylan
GH43	Candidate β-xylosidase/arabinosidase	133200	C6h, C12h, C24h	Xylan/Arabinoxylan
GH3	Β-xylosidase, bxl1	140746	B6h, B12h, B24h, C24h	Xylan/Arabinoxylan
GH11	Xylanase, xyn2	124931	B12h, B24h, C6h	Xylan/Arabinoxylan
GH30	Endo-β-1,4-xylanase, xyn4	90847	B6h, B12h, B24h, C6h	Xylan/Arabinoxylan
CE5	Acetyl xylan esterase, axe2	94461	C6h, C12h, C24h	Xylan/Arabinoxylan
CE5	Acetyl xylan esterase, axe1	139631	B24h	Xylan/Arabinoxylan
GH54	α-L-arabinofuranosidase, abf3	72252	C24h	Xylan/Arabinoxylan
GH74	Xyloglucanase, CEL74A	111943	B24h, C12h, C24h	Xyloglucan
GH16	Cell wall glucanase	96805	B6h, B12h, B24h, C6h, C12h, C24h	Carbohydrate transport and metabolism
GH27	α-D-galactosidase	75420	B12h	Carbohydrate transport and metabolism
GH3	Candidate β-N-acetylglucosaminidase	12475	B6h	Carbohydrate transport and metabolism
GH30	Candidate endo-β-1,4-xylanase	93498	B24h	Carbohydrate transport and metabolism
GH31	Candidate α-glucosidase	104546	B6h, B24h, C12h, C24h	Carbohydrate transport and metabolism
GH37	Candidate α,α-trehalase	102372	C6h, C12h, C24h	Carbohydrate transport and metabolism
GH65	Candidate α,α-trehalase	139320	B24h, C12h, C24h	Carbohydrate transport and metabolism
GH72	Candidate β-1,3-glucanosyltransferase	98936	C6h, C12h, C24h	Carbohydrate transport and metabolism
GT20	Bifunctional trehalose-6-phosphate synthase	72420	B6h, B12h	Carbohydrate transport and metabolism
GT20	Bifunctional trehalose-6-phosphate synthase	67350	B12h	Carbohydrate transport and metabolism
GT31	Distantly related to β-glycosyltransferases	101599	B12h	Carbohydrate transport and metabolism
GT35	Glycosyl transferase	23636	B6h, B12h, B24h, C6h	Carbohydrate transport and metabolism
GH16	Candidate glucanosyltransferase	66752	B6h, B12h, B24h, C6h, C12h, C24h	Cell wall biosynthesis and morphogenesis
GH72	Candidate β-1,3-glucanosyltransferase	9074	B6h, B12h, C6h, C12h, C24h	Cell wall biosynthesis and morphogenesis
GH72	Candidate β-1,3-glucanosyltransglycosylase	124639	B6h, B12h, B24h, C6h, C12h, C24h	Cell wall biosynthesis and morphogenesis
GT2	Glycosyl transferase	114628	B6h, B12h, B24h, C6h, C12h, C24h	Cell wall biosynthesis and morphogenesis
GT48	Candidate β-1,3-glucan synthase	139875	C6h, C12h	Cell wall biosynthesis and morphogenesis
GH18	Chitinase (chi18-5, chi46)	124526	C12h, C24h	Chitin
GH18	Chitinase (chi18-8)	130024	B12h	Chitin
GH20	N-acetyl-β-D-glucosaminidase, nag1	99285	C12h, C24h	Chitin
GH13	Candidate glycogen debranching enzyme	73564	B6h, B12h, B24h	Starch
GH15	Glucoamylase, gla	70185	C6h, C12h	Starch

**Table 2:** CAZymes detected in the secretome of *Aspergillus niger*.

CAZy Family	Predicted Protein	JGI Protein ID	Substrate/Time point	Possible Polysaccharide Substrate/Classification
GH17	Glucan endo-1,3-β-glucosidase(eglc)	158521	B6h, B12h, B24h, C6h, C12h, C24h	1,3–1,4-β-Glucan
GH55	Exo-β-1,3-glucanase (bxga, exgo)	156270	B12h, B24h, C6h, C12h, C24h	1,3–1,4-β-Glucan
GH55	β-1,3-exoglucanase	157838	B12h	1,3–1,4-β-Glucan
GH3	β-D-glucoside glucohydrolase M	163273	B6h, B12h, B24h, C12h	Cellulose
GH3	β-glucosidase 2	163842	B12h, B24h	Cellulose
GH3	β-glucosidase	165962	C24h	Cellulose
GH3	β-glucosidase	168801	B6h, B12h, B24h, C6h, C12h	Cellulose
GH5	Endo-β-1,4-glucanase A (egla)	156195	B6h, B12h, B24h, C12h, C24h	Cellulose
GH5	Endo-β-1,4-glucanase B (eng1)	161114	B6h, B12h, B24h, C6h, C12h, C24h	Cellulose
GH5	Endoglucanase B (eglb)	167967	B6h, B12h, C6h, C12h	Cellulose
GH5	Glucan 1,3-β-glucosidase A (exga)	168853	B6h, B12h	Cellulose
GH6	Exocellobiohydrolase	161440	B12h, B24h, C6h, C12h	Cellulose
GH6	β-glucancellobiohydrolase C	164557	B6h, B12h, B24h, C6h	Cellulose
GH7	Cellobiohydrolase B (cbhb)	156194	B6h, B12h, B24h, C6h, C12h, C24h	Cellulose
GH7	Cellobiohydrolase A (cbha)	161153	B6h, B12h, B24h, C6h, C12h, C24h	Cellulose
GH30	Glucan endo-1,6-β-glucosidase	158067	B6h, B12h, B24h, C6h, C12h, C24h	Cellulose
AA9 (GH61)	Endoglucanase IV	161785	B6h, B12h, C6h, C12h	Cellulose
AA9 (GH61)	Endoglucanase 4	166052	B24h	Cellulose
AA9 (GH61)	Putative endoglucanase IV	166976	B6h, B12h, B24h	Cellulose
CE8	Pectinesterase	157769	C6h, C12h, C24h	Pectin
CE8	Pectin methylesterase A (pmea)	158617	B6h, C6h, C12h	Pectin
CE8	Pectin methylesterase A (pmea)	159650	B6h, B12h, C6h, C12h, C24h	Pectin
CE12	Putative rhamnogalacturonan acetyl esterase	159617	C6h, C12h, C24h	Pectin
CE12	Rhamnogalacturonan acetyl esterase	162676	C6h, C12h	Pectin
CE16	Putative pectin acetylesterase	156782	C6h	Pectin
GH5	Endo-β-1,6-galactanase	158118	B6h, B12h, B24h, C6h, C12h	Pectin
GH28	Endopolygalacturonase-1	156180	C12h	Pectin
GH28	Endopolygalacturonase B (pgab)	157015	B6h, B12h, B24h, C6h	Pectin
GH28	Exopolygalacturonase (pgxb)	158660	C6h, C12h, C24h	Pectin
GH28	Endo-xylogalacturonan hydrolase A (xgha)	159651	B12h, B24h, C6h, C12h, C24h	Pectin
GH28	Endo-polygalacturonase D (pgad)	162788	C6h, C12h	Pectin
GH28	Exo-xylogalacturonan hydrolase (pgxa)	163648	C12h	Pectin
GH28	Rhamnogalacturonase (rhga)	164433	C12h	Pectin
GH28	Exopolygalacturonase X (pgax)	165048	C6h	Pectin
GH28	Rhamnogalacturonase B (rhgb)	166203	C6h, C12h	Pectin
GH28	Rhamnogalacturonan α-galacturonohydrolase	168924	C12h	Pectin
GH53	Arabinogalactan endo-1,4-β-galactosidas	169030	B24h, C6h	Pectin
PL1	Pectin lyase A (pela)	166220	B12h, C6h, C12h	Pectin
CE1	Feruloyl esterase A (faea)	162483	B6h, B12h, B24h, C6h, C12h, C24h	Phenylpropanoids
CE1	Feruloyl esterase C (faec)	164585	B6h, B12h	Phenylpropanoids
CE1	Feruloyl esterase (faeb)	165335	B6h, B12h, B24h, C6h, C12h, C24h	Phenylpropanoids
CE1	Acetyl xylan esterase (axea, acea)	164821	B24h, C6h, C12h	Xylan/Arabinoxylan
CE16	Acetylesterase	161113	B6h, B12h, C6h, C12h	Xylan/Arabinoxylan
GH3	Exo-1,4-β-xylosidase (xlnd)	156034	B6h, B12h, B24h, C6h, C12h	Xylan/Arabinoxylan
GH3	Bifunctional xylosidase-arabinosidase	168244	B24h	Xylan/Arabinoxylan
GH10	Endo-1,4-β-xylanase C	158107	B6h, B12h, B24h, C12h	Xylan/Arabinoxylan
GH11	Xylanase 2 / B (xynb, xlnb)	155137	B6h, B12h, B24h, C6h, C12h, C24h	Xylan/Arabinoxylan
GH11	Endo-1,4-β-xylanase B	166974	C6h	Xylan/Arabinoxylan
GH43	Arabinan endo-1,5-α-L-arabinosidase C	157571	B6h, B12h, B24h, C6h, C12h, C24h	Xylan/Arabinoxylan
GH43	Xylan β-xylosidase	161454	B12h, B24h	Xylan/Arabinoxylan
GH43	Glycosyl hydrolase family 43 protein	162327	B12h, B24h, C6h, C12h, C24h	Xylan/Arabinoxylan
GH43	Arabinan endo-α-1,5-L-arabinosidase A	162583	B6h, C6h, C24h	Xylan/Arabinoxylan
GH43	Endo-arabinase	166877	B12h	Xylan/Arabinoxylan
GH51	α-L-arabinofuranosidase A (abfa, exoa)	155097	C6h, C12h, C24h	Xylan/Arabinoxylan
GH51	α-L-arabinofuranosidase E	162554	B6h, B24h, C12h	Xylan/Arabinoxylan
GH54	α-L-arabinofuranosidase B	166753	B6h, B12h, B24h, C6h, C12h, C24h	Xylan/Arabinoxylan
GH62	α-L-arabinofuranosidase (axha)	158109	B6h, B12h, B24h, C6h, C12h, C24h	Xylan/Arabinoxylan
GH67	α-glucuronidase A	166362	B6h, B12h, B24h	Xylan/Arabinoxylan
GH35	β-galactosidase A (laca)	156240	B6h, B12h	Xyloglucan
GH12	Xyloglucan- endo-β-1,4-glucanase	155384	B6h, C6h, C12h	Xyloglucan
GH12	Endo-β-1,4-glucanase	158544	B6h, B12h, B24h, C12h, C24h	Xyloglucan
GH12	Endo-β-1,4-glucanase (egla)	166061	B6h, B12h, B24h, C6h, C12h, C24h	Xyloglucan
GH74	Xyloglucanase	155242	B12h, B24h	Xyloglucan
GH32	Invertase (suca, suc1)	162354	B24h, C6h	Carbohydrate transport and metabolism
GH32	Exo-inulinase E (inue, inu1)	165128	C6h, C12h, C24h	Carbohydrate transport and metabolism
GH65	α,αtrehalase	155210	B6h, B12h, B24h	Carbohydrate transport and metabolism
GH95	Glycosyl hydrolase	167353	B24h	Carbohydrate transport and metabolism
GH27	α-galactosidase II (aglb)/ melibiase	157631	B12h, C12h	Carbohydrate transport and metabolism
GH27	α-galactosidase A (ag1A;agla)	159990	B12h, B24h, C12h	Carbohydrate transport and metabolism
GH27	α-galactosidase D (agld)	165965	B6h, C12	Carbohydrate transport and metabolism
GH16	β-glucanase	155502	B24h, C24h	Cell wall biosynthesis and morphogenesis
GH16	Glycosidase crf1	156136	B6h, B12h, B24h, C6h, C12h, C24h	Cell wall biosynthesis and morphogenesis
GH16	GPI-anchored glucanosyltransferase	160973	B6h, C6h	Cell wall biosynthesis and morphogenesis
GH16	β-glucanase	163407	B6h, B12h, B24h, C6h, C12h, C24h	Cell wall biosynthesis and morphogenesis
GH17	1,3-β-glucanosyltransferase (bgt1)	161620	B6h, B12h, B24h	Cell wall biosynthesis and morphogenesis
GH72	1,3-β-glucanosyltransferase	156831	B12h, C12h	Cell wall biosynthesis and morphogenesis
GH72	1,3-β-glucanosyltransferase	161995	C6h	Cell wall biosynthesis and morphogenesis
GH72	1,3-β-glucanosyltransferase	162537	B6h, B24h, C6h, C12h, C24h	Cell wall biosynthesis and morphogenesis
GH72	1,3-β-glucanosyltransferase (gel1)	163189	B6h, B12h	Cell wall biosynthesis and morphogenesis
GH81	Glucan endo-1,3-β-D-glucosidase	155359	B12h, B24h	Cell wall biosynthesis and morphogenesis
GH13	Cell-wall 4-α-glucanotransferase (agta)	162772	B6h, B12h	Starch
GH13	Acid α-amylase (aama)	163584	B6h, B12h, B24h, C6h, C12h, C24h	Starch
GH15	Glucoamylase (glaa)	158641	B6h, B12h, B24h, C6h, C12h, C24h	Starch
GH18	Class V endochitinase (chib)	157223	B12h, B24h	Chitin
GH18	Exo-chitinase (cfci)	157878	B12h	Chitin
GH20	N-Acetyl-β-glucosaminidase (nag1)	162684	B24h	Chitin
GH5	β-mannanase (mana)	159852	B6h, B12h, B24h	Mannan
GH47	α-1,2-mannosidase	156279	B6h, B12h, B24h	Mannan
GH92	Glycosyl hydrolase	166207	B12h, B24h	Mannan

Compared to previous studies, we identified a higher number of CAZymes in *A*. *niger*. Adav *et al*. [20] identified 30 enzymes produced while culturing *A*. *niger* in a bioflo fermenter containing minimal medium and glucose. Oliveira *et al*. [33] identified 40 hydrolytic enzymes in the secretome of *A*. *niger* grown on a bioreactor containing minimal medium added of D-xylose (an inducer of cellulases and hemicellulases) or D-maltose as the sole carbon sources. However, in those reports, the inducers were simple carbon sources, and not complex carbon sources such as sugarcane bagasse and culm. An exception is the work from Souza *et al*. [25] that used SEB as carbon source, but they identified only 17 proteins using a much less refined proteomics method.

Interestingly, only one enzyme was reported in common to all these studies, the acid α-amylase (AamA). This enzyme belongs to the GH13 family and it cleaves internal α-(1,4)-glycosidic bonds in starch and glycogen [34]. However, we identified the most important enzymes related to biomass degradation. To hydrolyze the cellulose chains into monomers, the main chain must be cleaved internally, and this event is performed by endoglucanases (GH5, GH12). Likewise, the release cellobiose occurs via the action of exoglucanases (GH6, GH7), and it is subsequently converted into glucose by β-glucosidases (GH3) [35]. The hemicellulose fraction is formed by arabinoxylan, β-glucan and xyloglucan, and due to this variety of substrates, the enzymatic mixture required to break it down is more diverse, including endoxylanases (GH10, GH11), β-xylosidase (GH3), arabinofuranosidases (GH43, GH51, GH54), galactosidases (GH35) and others [22]. In the *A*. *niger* secretome, all of these enzymes were present at the earliest time point examined in this study (6 h).

We observed some differences for *T*. *reesei* grown on other carbon sources since the number of *T*. *reesei* CAZymes was higher than that reported by Herpoël-Gimbert *et al*. [22]; they identified 22 hydrolytic enzymes using lactose and xylose as carbon sources. However, Adav *et al*. [24] identified over 90 CAZymes using a quantitative proteomic approach, the iTRAQ system. Moreover, a recent study using untreated sugarcane bagasse investigated the secretome of two *Trichoderma* strains using solid-state fermentation [26]. In this study, they identified 39 GHs, and other proteins that play an important role in biomass degradation (for example, swollenin). These differences could be due either to the carbon source, culture conditions or experimental design. However, in contrast to *A*. *niger*, the most important proteins found in this study were also present in the afore mentioned reports, such as cellobiohydrolases (Cbh1/Cbh2), endoglucanases (Eg1, Eg2 and Eg4) and β-glucosidases (Bgl1/Bgl2). We also identified hemicellulases involved in the cleavage of the main chain of xylan and xyloglucan (endoxylanase and xyloglucanase) and the side chain of hemicelluloses, including β-xylosidase, α-arabinofuranosidase and α-galactosidase, and acetylxylan esterase.

### Differences in the enzyme secretion in distinct sugarcane biomass

**Fig 3:**
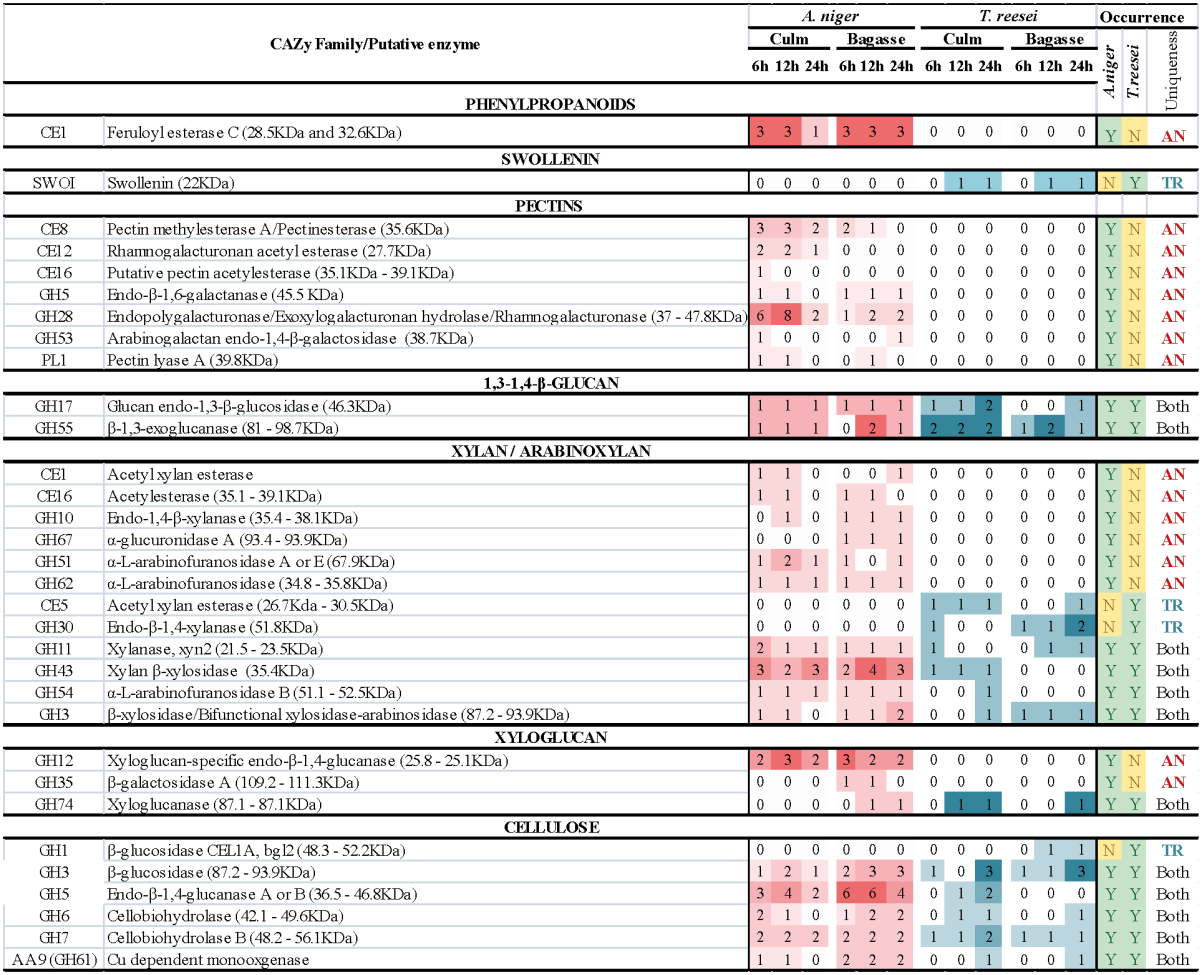
Graphical representation of secreted CAZYmes. Heat-map of the number of enzymes of each CAZY family secreted by *A*. *niger* and *T*. *reesei* after 6, 12 and 24 hours (h) growth on sugarcane culm and bagasse. This map includes only enzymes/proteins related to biomass degradation (Tables 1 and 2).

An additional goal of this study was to better understand the differences in enzyme patterns in distinct biomass types. Thus, in addition to bagasse, culm *in natura* was used as a sole carbon source. Fig 3 shows a heat-map representing the number of enzymes identified for each class of enzymes (CAZymes classification was used) in each family of glycosyl hydrolase directly related to biomass degradation (see Tables 1 and 2) in *A*. *niger* and *T*. *reesei* after 6, 12 and 24 hours growing on untreated sugarcane culm and pretreated bagasse.

The pattern of enzyme production observed for *A*. *niger* was similar for culm and bagasse. However, analyzing the enzymes secreted exclusively when *A*.*niger* was grown on culm, we observed a range of pectinases (Fig 3, Table 2), which have a variety of biotechnological and industrial applications, and several of them have been cloned, characterized, or have had their production optimized [12, 36–38]. Although seven pectinases were identified as being secreted using both carbon sources, twelve pectinases were secreted exclusively when *A*.*niger* was grown on culm (Fig 3, Table 2). It is likely that the fraction containing more soluble pectins was lost during the sugarcane crushing (probably because of the hot water treatment), rendering bagasse with proportionally less pectin compared to culm. Therefore, the induction of these enzymes is higher when the fungi are grown using culm as a carbon source. Curiously, although *T*. *reesei* secreted several enzymes when growing on culm and bagasse, none were related to pectin, even though its genome encodes several pectinases.

**Fig 4:**
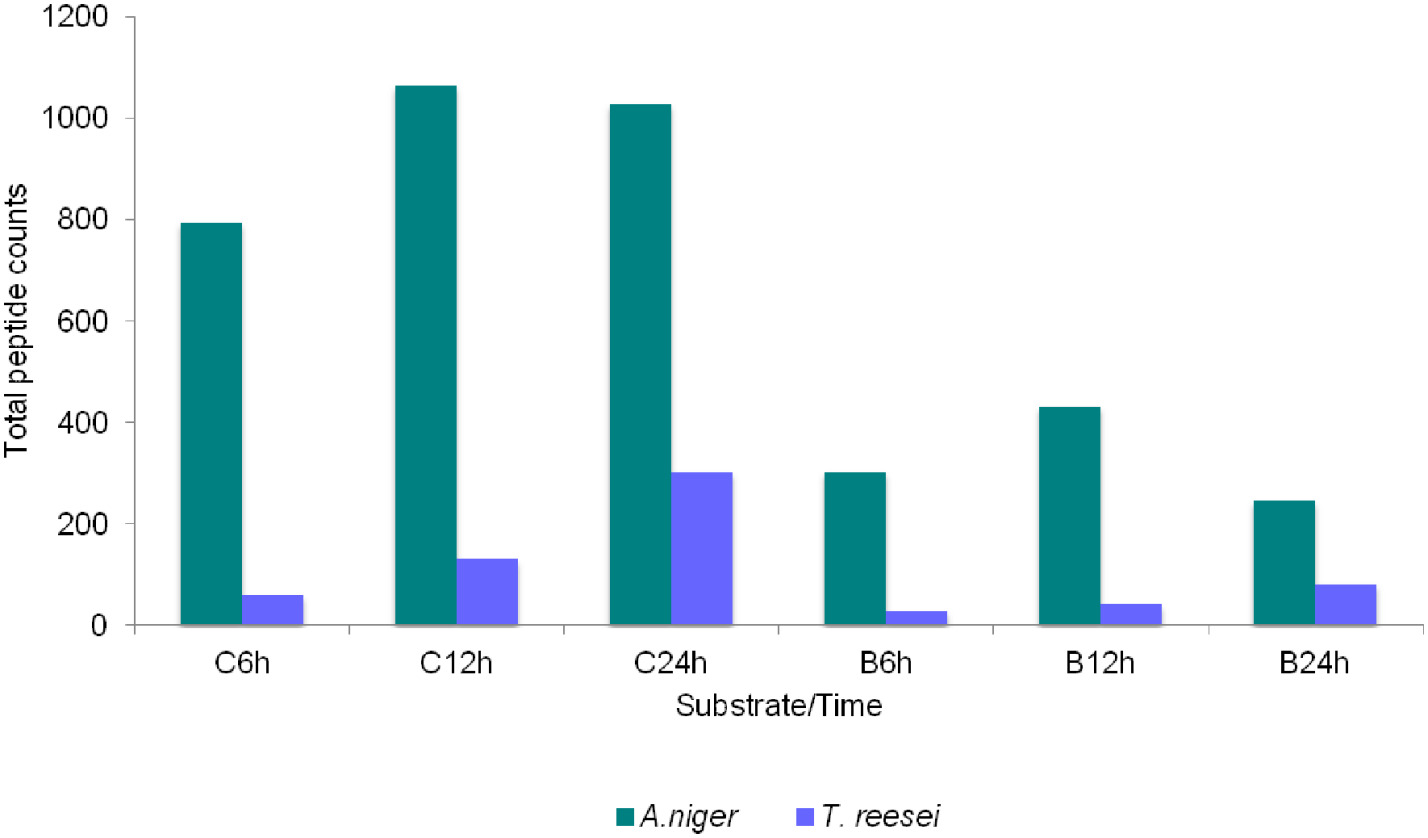
A comparison of the abundance of secreted enzymes by *A*. *niger* and *T*. *reesei*. A semi-quantitative analysis of the amount of enzyme secreted by both fungi after 6, 12 and 24 hours (h) growth on sugarcane culm (C) and bagasse (B).

Despite the variety of enzymes produced by both fungi, a comparative analysis of the total number of peptides, which may be indicative of protein/enzyme abundance [39, 40] (Fig 4), suggested that *A*. *niger* and *T*. *reesei* secreted more enzymes when grown on culm. As mentioned previously, there were no differences in fungi growth between the two substrates (Fig 2). Thus, the higher abundance of peptides produced by fungi growing on culm correlates with the greater amount of sugars in the supernatant (Fig 1b and 1d). This result could be related to differences in the recalcitrance of substrates, which reflects the different levels of complexity of the cell walls, as culm did not receive any pretreatment. However, how fungi sense these differences remains unclear.

### Comparison between *T*. *reesei* and *A*. *niger* secretomes

The secretome profiles of these two fungi were compared, and our data indicates they differed considerably (Fig 3, Tables 1 and 2). As mentioned before, the assembly, modification and breakdown of complex carbohydrate and glycoconjugates are carried out by the CAZymes [41]. However glycosyl transferases and the carbohydrate esterases from the family CE10 genes are not involved in degradation of plant cell walls. In the *A*.*niger* supernatant, we detected 65 enzymes directly related to biomass degradation, approximately 22% of the total of carbohydrate-active enzymes encoded by its genome (292 proteins, excluding GTs), whereas *T*. *reesei* secreted 24 enzymes (approximately 10% of its 228 CAZymes, excluding GTs). GHs found exclusively in the *A*. *niger* secretome were related to hemicellulose degradation, such as GH10 (endo-1,4-β-xylanase), GH51 and GH62 (both α-L-arabinofuranosidases), and pectin, including endopolygalacturonase (GH28) and endo-1,4-β galactosidase (GH53) (Fig 3). Important esterases were also present in *A*. *niger*, but not *T*. *reesei*, such as the acetyl xylan esterase (CE1) and acetylesterase (CE16), that catalyzes the hydrolysis of acetyl groups from hemicellulose, and pectin methyl esterase (CE8), acetyl esterase (CE12), that have activity against pectin.

*T*. *reesei* produces the most commercially used cellulases [42], and among the GHs related to biomass degradation found exclusively on its secretome, we identified a β-glucosidase from the GH1 family. At earlier time points, the mode by which *T*. *reesei* attacks arabinoxylans seems to differ from that of *A*. *niger*, with an exclusive acetyl xylan esterase (CE5) and an endo-xylanase (GH30). No evidence of the presence of laccases were found for any of the species studied, suggesting that the hydrolytic attack of these fungi toward sugarcane biomass does not involve lignin degradation, at least on the time points tested.

We also performed a comparative analysis of the total number of peptides. In agreement with previous studies [23, 24], the cellobiohydrolase Cel7A displayed the highest number of peptides in the *T*. *reesei* secretome (S1 Table). The cellobiohydrolase Cel6A, was the third most abundant enzyme; together with CEl7A, these enzymes represent 80% of the peptides from cellulases (S1 Table), as reported by other authors [22, 24]. An enzyme from the GH16, a putative glucanosyl transferase (ID 66752) related to cell wall biosynthesis and morphogenesis, was the second most abundant protein. Unlike *T*. *reesei*, the relative analysis of abundance revealed that the peptides from α-L-arabinofuranosidase B (GH54) and xylanase 2 (GH11) were the most abundant peptides in *A*. *niger*. Although Adav *et al*. [20] described the same arabinofuranosidase as the one of most abundant proteins after D-xylose induction, xylanase 2 was barely detected. This xylanase was induced by culm and bagasse in *A*. *niger*, but it was less prevalent in *T*. *reesei* (Fig 3, S1 Table).

**Fig 5:**
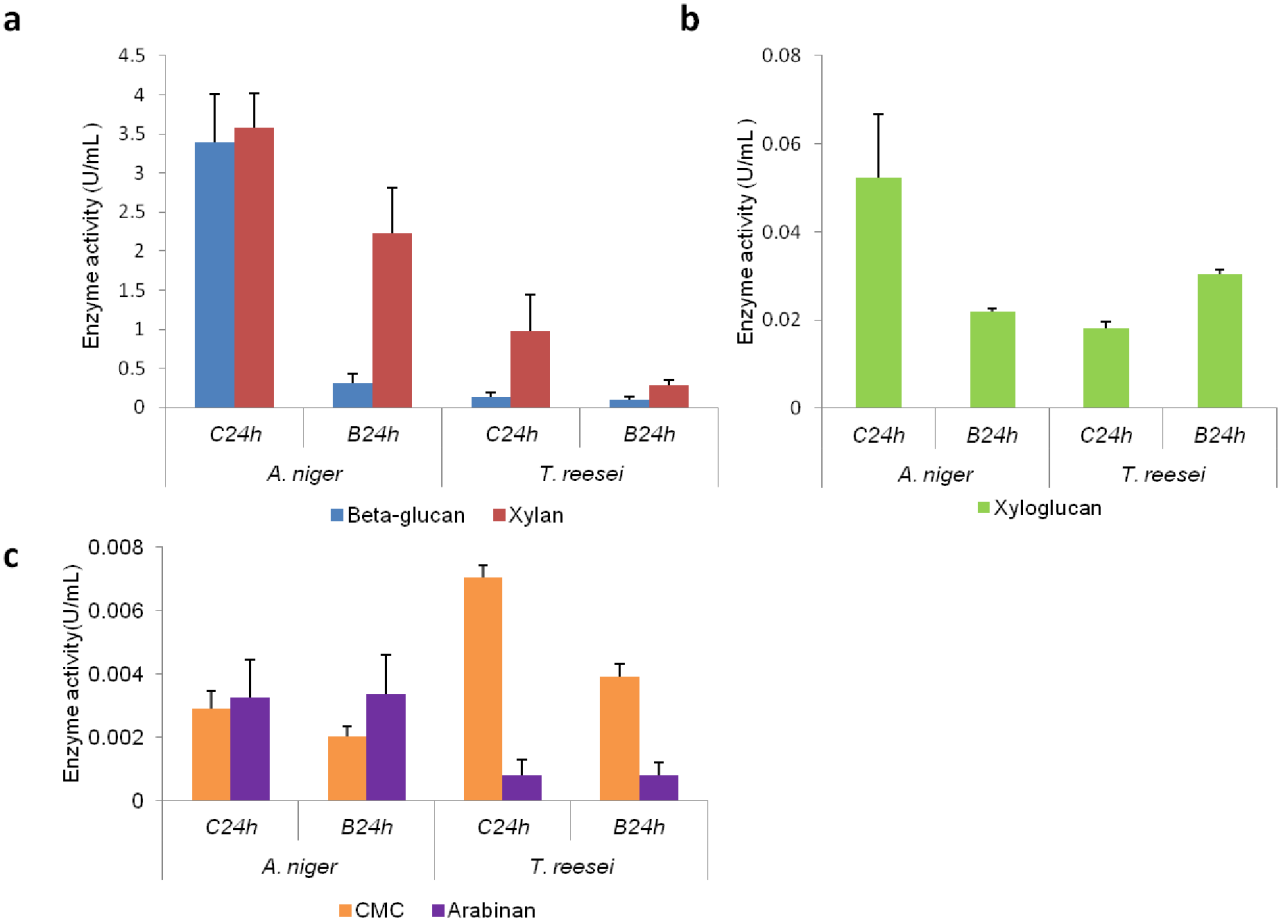
Estimation of enzyme activities. Enzymatic activities (U/mL) against different substrates of *A*. *niger* and *T*. *reesei* after 24 hours (h) growth on sugarcane culm (C) and bagasse (B). a) B-glucan and xylan b) Xyloglucan, and c) CMC and Arabinan. Each bar represents the mean and the standard deviation of values from three independent experiments.

Interestingly, when we compared the growth of both fungi, *T*. *reesei* had a larger mycelia mass compared to *A*. *niger* (indirectly measured by nitrogen content, Fig 2). Considering the abundance of secreted enzymes in both fungi, *A*. *niger* produced not only a wider range of enzymes but also secreted higher quantities compared to *T*. *reesei* (Fig 4). To verify whether the abundance of peptides reflected the abundance of enzymes, we performed enzymatic assays using beechwood, β-glucan, debranched arabinan from sugar beet, xyloglucan from tamarind and CMC as substrates. Given the low sensitivity of the assay and the small amount of protein, we measured the enzymatic activities at 24 hours, the time point with the highest enzyme production (Tables 1 and 2). Supernatants derived from both cultures were capable of hydrolyzing hemicelluloses, such as β-glucan, xylan (Fig 5a), xyloglucan (Fig 5b), arabinan (Fig 5c), and cellulose: CMC (Fig 5c), which is in agreement with the identified enzymes. Moreover, enzymes derived from the supernatant fraction of *A*. *niger* were able to hydrolyze the majority of substrates more effectively than *T*. *reesei*, corroborating the peptide proportion data presented in showed in Fig 4. Furthermore, enzymatic activity toward CMC was higher in *T*. *reesei*, likely due to its remarkable cellulolytic ability.

**Fig 6:**
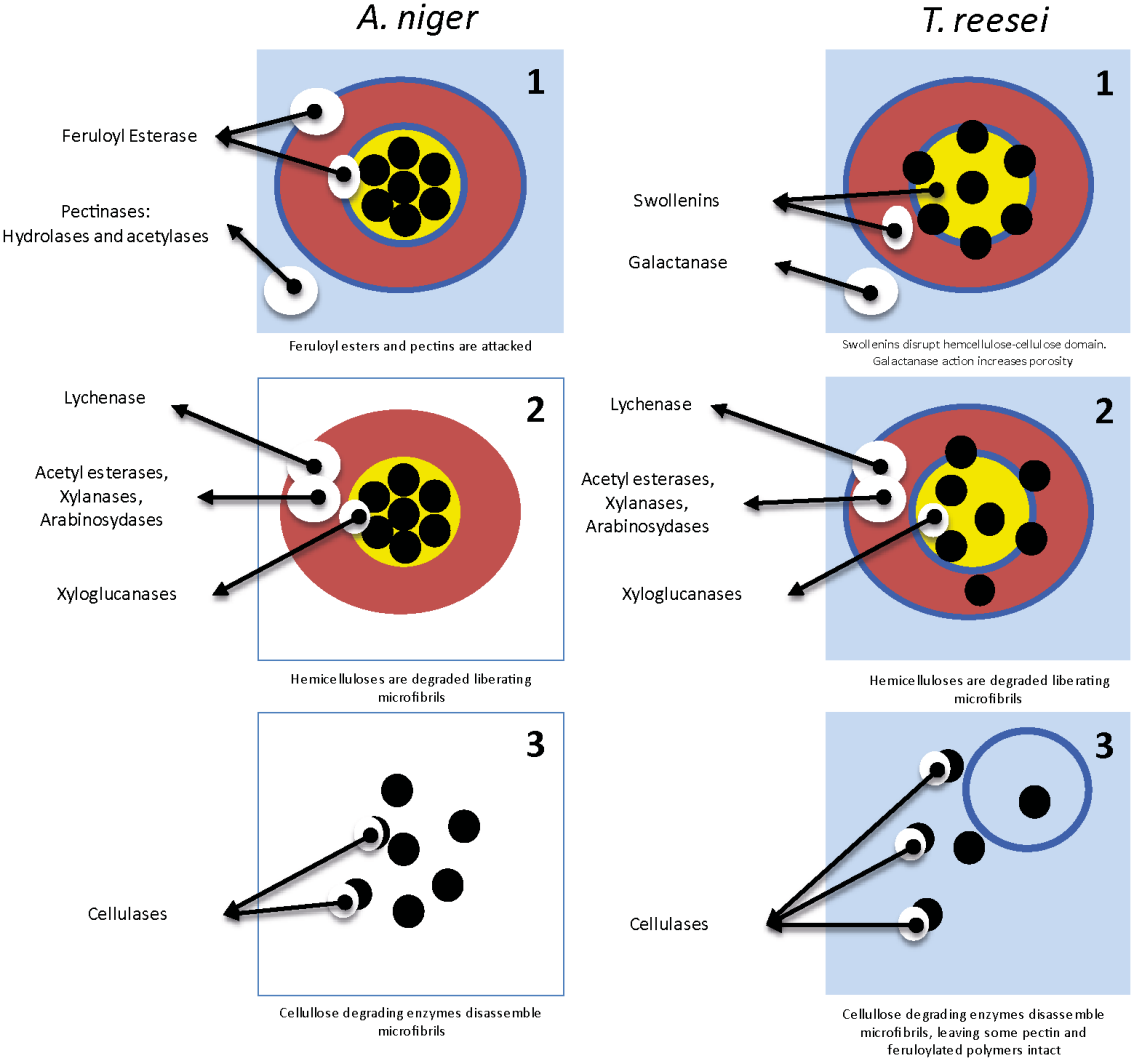
Schematic representation of hypothetical modes of attack of the enzyme complexes produced by *A*. *niger* (left) and *T*. *reesei* (right). One cell wall architectural unit of sugarcane (De Souza et al., 2013) is represented. Light blue: pectins; dark blue: feruloyl esters; red: hemicelluloses—beta-glucan and arabinoxylan; yellow: xyloglucan; black: cellulose microfibrils. Cell wall degradation is schematically represented in three steps for each fungi attack so as to compare the two different strategies hypothesized in this work. Whereas *A*. *niger* degrades cell wall components of different classes with approximately the same intensity, including action on feruloyl esters, pectins, hemicelluloses and cellulose, *T*. *reesei* maximizes penetration into the cell wall matrix, lacking feruloyl esterases, having limited action on pectins, but disassembling more efficiently the cellulose-xyloglucan network and attacking mainly the cellulose microfibrils.

Therefore, we evaluated secreted enzymes involved in cell wall degradation by both fungi over the experimental time course and tried to correlate the monosaccharides released in the supernatant and enzymes related to the degradation of their polysaccharides. As we mentioned before, our data suggests that arabinoxylan is been consumed within the first 6 hours after attack by both fungal species. Hypothetically, at least for *A*. *niger*, the attack of feruloyl esterases and the acetyl esterases might allow for arabinoxylan degradation. At early time points, the general performance of the enzyme production system of *A*. *niger* corroborated the model of sugarcane cell wall hydrolysis proposed by De Souza *et al*. [4] that reported that sugarcane cell walls are composed of domains that can be extracted with progressively higher concentrations of alkali. Pectins (ca. 10% of the wall) are more soluble, followed by 1,3 and 1,4-β-glucans (ca.10% of the wall) and highly interactive arabinoxylans (ca. 40% of the wall) and a xyloglucan/xylan-cellulose domain (ca. 40% of the wall) that is less soluble. Thus pectinases and esterases should be the first enzymes to act on the walls, opening the way for the hemicellulases and cellulases (Fig 6). Observing the appearance of enzymes related to biomass degradation (Fig 3), we found that the carbohydrate esterases (feruloyl and acetyl xylan esterases (CE1), pectin methylesterase (CE8), rhamnogalacturonan acetyl esterase (CE12), pectin and acetyl esterase (CE16)) were some of the first enzymes secreted by *A*. *niger*. These enzymes have molecular weights below the threshold that is considered the pore size of the cell wall, 35–40 Angstrons [43]. Thus, these enzymes are likely to penetrate the cell wall matrix before other glycosyl hydrolases that will attach polymer decorations and main chains. Thus, by deferuloylating, demethylating and deacetylating pectins and xylans, the hydrolytic system would hydrate the wall environment (due to the lack of hydrophobic branches), while also making the main chains of these polymers more accessible to endo-enzymes.

From the proteomics point of view, *T*. *reesei* displays a different strategy to disassemble sugarcane cell walls compared with *A*. *niger* (Fig 6). The former species produced relatively low quantities and variety of enzymes, and it did not produce pectinases at all. The fact that no feruloyl esterase is present in *T*. *reesei* extracts suggests that this species employs a mechanism that uses proportionally fewer debranching enzymes in the early stages of biomass degradation and that it is able to attack cellulose microfibrils without a prior attack to the phenylpropanoids of the cell wall. On the other hand, swollenin was detected in *T*.*reesei* after 12 hours in culm and bagasse. This swollenin isolated from *T*.*reesei* (the protein was named SWO1) behaves like a plant expansin. When purified, SWO1 disrupted the cell wall structure without the production of free glucose [44]. Swollenin and the acetyl-xylan-esterase (CE5–30.5 KDa), which were present only in the *T*. *reesei* genome and secretome (Fig 3, Table 1), likely act by disrupting the cell wall architecture and loosening polymer-polymer interaction in a way that polysaccharides become more accessible to glycosyl hydrolases, such as 1,3–1,4-β-glucanases, arabinoxylanases and cellulases (Fig 6).

Despite the distinct modes of attack to the biomass, both fungi are able to break down the biomass cell wall since the earliest time points, but we can suggest that *A*. *niger* invests more in cell wall hydrolysis in terms of number of enzymes and enzyme activities related to cell wall hydrolysis than *T*. *reesei*, which secreted fewer enzymes. These two different behaviors are probably associated with the biology of each species. Another important aspect that seems to influence the observed behavior of both fungi is that they are limited, to a certain extent, by their respective genomes, i.e., the enzyme arsenals to address biomass [15, 45]. Notwithstanding, it must taken into consideration that *T*. *reesei* was submitted to several rounds of random mutagenesis to obtain the hypercellulolytic strain RUT-C30.

## Conclusions

Here, we provide the first comparative secretome analysis of the most important lignocellulolytic fungal species, *A*. *niger* and *T*. *reesei*, growing on sugarcane biomass. This secretome study indicates that biomass degradation begins within the first 6 hours of fungal growth. A proteomic approach was used to analyze the secretome profiles of *A*. *niger* and *T*. *reesei*, and our data indicated that the two fungal species have different modes of attacking the same biomass, at least within 24 hours of the saccharification process. Thus, we advanced our understanding of the synergic mode of attack of swollenin, esterases, and glycosyl hydrolases in the context of enzyme cocktails and architecture of the plant cell wall. Using a semi-quantitative method based on peptide counts, we estimated the relative differences in the amount of extracellular enzyme production. We noticed that the induction of hydrolytic enzymes is higher when both fungi were growing using culm as a carbon source, probably due the higher recalcitrance of this substrate. At the time points measured, *A*. *niger* produced more enzymes (quantitatively and qualitatively) than *T*. *reesei*, but both species were able to disassemble the carbohydrate portion of sugarcane cell walls. Considering that *T*. *reesei* and *A*. *niger* have different mechanisms for degrading biomass, these data suggest that a combination of enzyme from the two species might be an interesting option to increase saccharification efficiency. In other words, the two fungal species might be combined for use in industrial processes.

## Supporting Information

S1 Fig SDS-PAGEs of the secretome of A. *niger* and *T*. *reesei* grown on sugarcane biomass. Proteins secreted by *A*. *niger* (A) and *T*. *reesei* (B) after 6, 12 and 24 hours (h) growing on sugarcane culm (C) and bagasse (B). Lane M: molecular weight marker.
(TIF)

S1 Table CAZymes identified in the secretome of *A*. *niger* and *T*. *reesei*. This file includes all hydrolytic enzymes identified in the secretome of *A*. *niger* and *T*. *reesei*, their respective molecular weights, number of identified peptides, peptide sequences, mass-to-charge ratios (m/z), number of unique peptides, and presence/absence of signal peptide.
(XLSX)

S2 Table Proteins with signal peptide (not CAZymes) identified in both secretomes. This file includes other proteins identified in the secretome of *A*. *niger* (A) and *T*. *reesei* (B), their respective molecular weights, number of identified peptides, peptide sequences, mass-to-charge ratios (m/z) and number of unique peptides.
(XLSX)
