# Supplementary figures and images for: Comparative Secretome Analysis of Trichoderma reesei and Aspergillus niger during Growth on Sugarcane Biomass
Source: PLoS One. 2015 Jun 8;10(6):e0129275. doi: 10.1371/journal.pone.0129275 (PMC4460134; doi:10.1371/journal.pone.0129275)

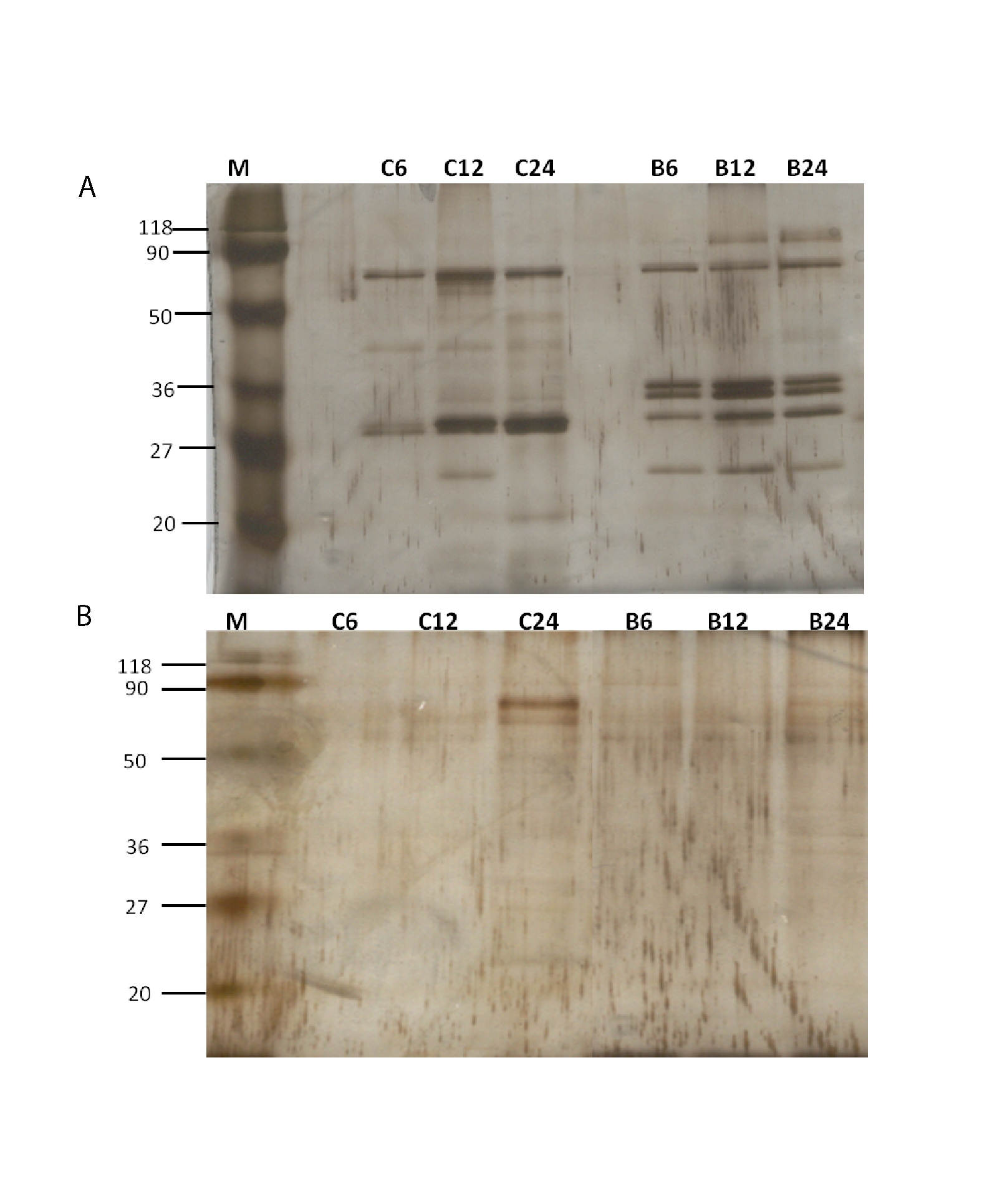

Supplement: S1 Fig — Proteins secreted by A. niger (A) and T. reesei (B) after 6, 12 and 24 hours (h) growing on sugarcane culm (C) and bagasse (B). Lane M: molecular weight marker. (TIF) [file pone.0129275.s001.tif]
